# Methodology for enabling high-throughput simultaneous saccharification and fermentation screening of yeast using solid biomass as a substrate

**DOI:** 10.1186/s13068-014-0181-z

**Published:** 2015-01-22

**Authors:** Adam Elliston, Ian P Wood, Marie J Soucouri, Rachelle J Tantale, Jo Dicks, Ian N Roberts, Keith W Waldron

**Affiliations:** The Biorefinery Centre, Institute of Food Research, Norwich Research Park, Colney, Norwich, NR4 7UA UK; École supérieure d’ingénieurs Réunion Océan Indien, Génie Biologique, Université de La Réunion, Parc Technologique Universitaire, 2 Rue Joseph Wetzell, 97490 Sainte-Clotilde, La Réunion France; Institut Universitaire de Technologie, Universite de la Reunion, 40 avenue de Soweto, BP 373, 97455 Saint-Pierre Cedex, La Réunion France; The National Collection of Yeast Cultures, Institute of Food Research, Norwich Research Park, Colney, Norwich, NR4 7UA UK

**Keywords:** High-throughput screening, Enzyme saccharification, Simultaneous saccharification and fermentation, Cellulosic biomass, Cellulosic ethanol, Biorefining, Biomass

## Abstract

**Background:**

High-throughput (HTP) screening is becoming an increasingly useful tool for collating biological data which would otherwise require the employment of excessive resources. Second generation biofuel production is one such process. HTP screening allows the investigation of large sample sets to be undertaken with increased speed and cost effectiveness. This paper outlines a methodology that will enable solid lignocellulosic substrates to be hydrolyzed and fermented at a 96-well plate scale, facilitating HTP screening of ethanol production, whilst maintaining repeatability similar to that achieved at a larger scale.

**Results:**

The results showed that utilizing sheets of biomass of consistent density (handbills), for paper, and slurries of pretreated biomass that could be pipetted allowed standardized and accurate transfers to 96-well plates to be achieved (±3.1 and 1.7%, respectively). Processing these substrates by simultaneous saccharification and fermentation (SSF) at various volumes showed no significant difference on final ethanol yields, either at standard shake flask (200 mL), universal bottle (10 mL) or 96-well plate (1 mL) scales. Substrate concentrations of up to 10% (w/v) were trialed successfully for SSFs at 1 mL volume. The methodology was successfully tested by showing the effects of steam explosion pretreatment on both oilseed rape and wheat straws.

**Conclusions:**

This methodology could be used to replace large shake flask reactions with comparatively fast 96-well plate SSF assays allowing for HTP experimentation. Additionally this method is compatible with a number of standardized assay techniques such as simple colorimetric, High-performance liquid chromatography (HPLC) and Nuclear magnetic resonance (NMR) spectroscopy. Furthermore this research has practical uses in the biorefining of biomass substrates for second generation biofuels and novel biobased chemicals by allowing HTP SSF screening, which should allow selected samples to be scaled up or studied in more detail.

## Background

Second generation bioethanol production involves a number of consecutive stages, each with a multitude of combinations. Broadly broken down into pretreatment, hydrolysis, fermentation and distillation and/or separation, the overall process for any given substrate could potentially have thousands of different permutations.

In order to ascertain the most effective, economic or rapid way to produce a final product, screening various process parameters may be required. With this in mind, rapid screening of biomass as far as the hydrolysis stage has been established [1-4]. However further research is needed to take this process beyond fermentable sugar yields, to include the effect of yeast cultures, or indeed other micro-organisms. Of particular interest is the potential effect of fermentation inhibitors released from the biomass during processing on final alcohol yields, which may be process or substrate-specific [5].

Both separate hydrolysis and fermentation (SHF) and simultaneous saccharification and fermentation (SSF) methodologies are important. However the current paradigm is focused more towards SSF [6]. This is due to the simplification of the engineering requirement (decreased capital and operational expenditures) and the reduced potential for microbial contamination prior to the addition of yeast.

In the case of second generation biofuels, more realistic results could be obtained if solid, ‘real world’ substrates were used. However this adds its own unique set of problems to high-throughput (HTP) screening. One such difficulty is reliably, repeatedly and rapidly dosing small quantities of solid material. Hand weighing is too labor intensive and time consuming, and is therefore impractical for HTP screening. Current methods for overcoming this problem in relation to saccharification alone include using automatic weighing equipment [7], handbills (sheets of dry biomass which can be cut into repeatable weights) [8] and biomass slurry pipetting [4]. Further major obstacles include evaporation of samples, with the small scales utilized in HTP (typically ≤1 mL) being highly susceptible. Moreover, mixing can become problematic at smaller scales.

This paper aims to address these difficulties and offers a solution allowing for effective screening of these notoriously difficult substrates [3,4].

## Results

HTP screening is dependent on the speed and automation of all the individual stages that constitute the overall process. One of the main difficulties that needs to be addressed when using solid substrates is the necessity of accurately and repeatedly weighing samples in as short a time frame as possible. The three main methods currently in use for saccharification studies involve (1) utilizing fully automatic weighing apparatus [7], (2) drying the material into thin handbills of regular thickness and weight and then punching out discs [8] and (3) making slurries of the material in order to allow it to be pipetted with standard liquid handling devices [4]. Automatic weighing requires specialist equipment that may not be available to most laboratories. Again, handbill creation also generally requires additional equipment. Nevertheless, a number of widely accepted assays use solid filter paper as substrate, such as the filter paper unit (FPU) measurement of cellulase activity [9]. Slurry pipetting techniques are perhaps the most accessible techniques for most laboratories, as they can be carried out with either manual or automated full liquid handling systems, and these have been applied in this study for HTP SSF analyses using a 96-well plate format suitable for robotics.

**Figure 1:**
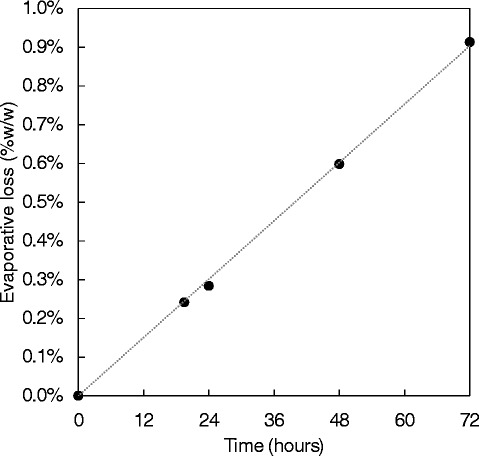
**Evaporative loss from plate incubated at 50°C for 72 hours.** Evaporative loss is shown as a percentage of original mass. Sample replicates n = 96. Error bars are not included as they are too small to be clearly seen on the figure.

Another challenge concerning HTP screening using small scale incubations is that of evaporative loss [8]. In order to quantify the loss, a 1.0 mL matrix storage tube plate was dried to a constant weight at 50°C, weighed, and then each well was filled with 1.0 mL yeast nitrogen base (YNB; Formedium, Hunstanton, United Kingdom). The plate was then incubated at 50°C (enzyme optimum temperature) over 72 hours and weighed at 24, 48 and 72 hour time points (Figure 1), showing minimal evaporative losses of 0.28%, 0.60% and 0.91%, respectively. The low rate of evaporation can also be seen to be linear, with an R^2^ value of 0.9991.

### The effect of volume on solid substrate simultaneous saccharification and fermentation using handbill-style weighing methodology

Initially, HTP methodology was explored using Whatman number one filter paper (FP) and office copier paper (OCP) substrates which could be weighed accurately and rapidly. The regularity of thickness and density of these substrates, along with the repeatability of paper disc creation, permit the simple and rapid allocation of substrate to small matrix tubes. A similar method is employed in the use of handbills [8] and also in the measurement of cellulase activity [9-11]. In this case, a standard area of FP is considered to have a replicable mass due to the assumption of consistent paper thickness. FP- and OCP-punched discs (6 mm in diameter) were found to have highly repeatable masses; 2.36 mg ±3.1% (w/w) and 2.19 mg ±2.5% (w/w), respectively, where n = 6.

In order to compare a 96-well plate mini-tube SSF approach with larger scale methodologies, comparative experiments were carried out using standard shake flask methods [12-15]. These involved performing SSF in 200 mL volumes (in 500 mL Erlenmeyer flasks), 10 mL volumes (in 30 mL screw-capped culture bottles) and 1 mL volumes (in 1 mL screw-cap matrix storage tubes for 96-well plates). Previous experimentation (results not shown) demonstrated the potential of using 96-well plates to screen enzymatic hydrolyses of solid substrates [1-4] at a small scale (approximately 1 mL).

**Figure 2:**
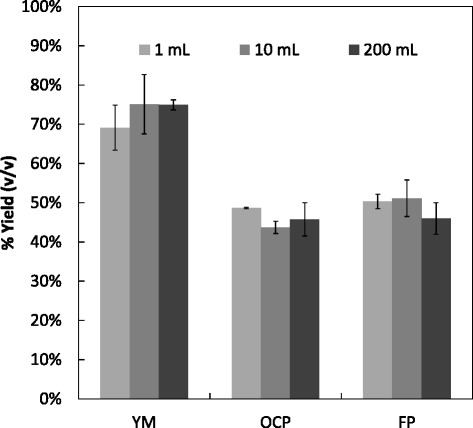
**Percentage ethanol yield at various vessel volumes.** Ethanol yields based on theoretical maximum production. Handbill-style biomass weighing over 200 mL, 10 mL and 1 mL vessel volumes. Samples FP: Filter paper (Whatman number one), OCP: Office Copier Paper, YM: Yeast and Mould Media. Sample replicates n = 3. OCP and FP were included at 2.5% (w/v) dry matter along with cellulase at 20 FPU/g; YM contained glucose at 0.9% w/v. Samples were incubated for 72 hours at 25°C.

A comparison of SSF at the three volumes (1, 10 and 200 mL) was conducted using yeast and mould media (YM) containing glucose at 0.9% (w/v), OCP and FP (each at substrate loadings of 2.5% w/v). Samples were incubated at 25°C for 72 hours on a single rotating shaker. Details of enzyme and yeast addition are given in the Materials section. The results (Figure 2) showed the level of ethanol produced as a percentage of theoretical ethanol yields calculated from glucose (YM) or cellulose contents given by sugars analysis (78.07% for FP; 53.09% for OCP). No statistical difference in ethanol yield was observed between flask sizes using either YM (*F*_2,6_ = 1.73, *P* = 0.288), OCP (*F*_2,6_ = 0.49, *P* = 0.642) or FP (*F*_2,6_ = 1.56, *P* = 0.297). Sample size n = 3 in all cases.

The results showed that for each substrate, the percentage yield of ethanol was similar at each volume scale. Hence, for pure cellulose or glucose, the HTP small scale method is highly effective and, in its own right, provides a useful approach for screening yeasts in an SSF environment.

### Finely milled pretreated biomass slurry as a substrate for simultaneous saccharification and fermentation screening

In order to develop and evaluate the HTP method using more relevant pretreated lignocellulose biomass, the potential for rapid and accurate allocation of substrate was tested using a finely divided, pretreated oilseed rape straw slurry (OSRS). Aliquots (1 mL, 1% w/v) of OSRS were transferred by a Tecan Freedom Evo™ liquid handling robot equipped with wide bore tips to pre-weighed 1 mL matrix tubes held in a 96-well plate format. The slurry was then dried to a constant weight and the tubes weighed again to infer the amount of dry solid. Across an entire 96-well plate, the average mass of 12.97 ± 0.22 mg (1.7% w/w) was transferred to each well (n = 96).

**Figure 3:**
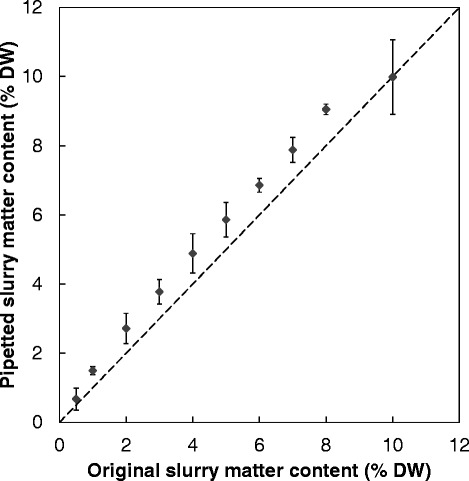
**Comparison of efficacy in pipetting biomass slurry at a range of dry matter contents.** Dashed diagonal line represents unity. Sample replicates n = 3. DW, dry weight.

The effectiveness of this approach was tested further by assessing the transfer of aliquots containing higher substrate concentrations. The results showed that OSRS containing between 0.5 and 10% dry matter (w/v) could be repeatedly and accurately transferred to the individual tubes of a 96-well plate (Figure 3; n = 3). Therefore, the use of biomass slurries allows the use of automated liquid transfer or, where this is not available, also facilitates the use of standard multi-pipettes.

### Effect of lignocellulose solids concentration on simultaneous saccharification and fermentation capacity

Having established a tractable methodology for rapidly and accurately transferring solid lignocellulose substrate into matrix tubes, a further comparative SSF study was carried out to compare ethanol production at the 1 mL and 10 mL scales. In order to move the approach closer to conditions relevant to biomass exploitation, the study also compared SSF of OSRS at 2.5% (w/v) and 10% (w/v), and with YM.

**Figure 4:**
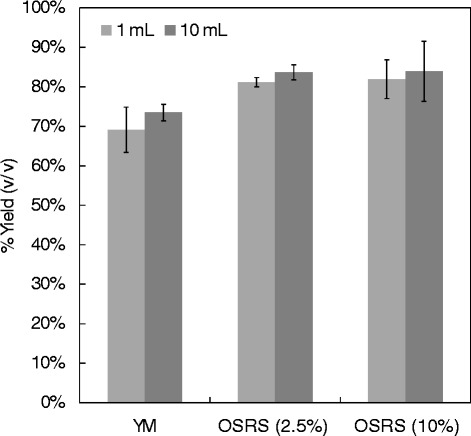
**Comparison of ethanol yield during simultaneous saccharification and fermentation of pretreated biomass at different vessel volumes.** Yields based on theoretical ethanol maximum production, oilseed rape straw (OSRS) slurry 2.5%, 10% substrate concentration and yeast and mould media (YM) substrates. Sample replicates n = 3.

The results (Figure 4) showed that at 1 mL and 10 mL scales, the SSF production of ethanol (as a percentage of theoretical yield) showed no significant difference at both volumes. Furthermore, the OSRS substrate loadings of 2.5% (w/v) and 10% (w/v) gave similar results (one-way analysis of variance (ANOVA) YM (*F*_1,4_ = 1.32, *P* = 0.314); OSRS 2.5% (*F*_1,4_ = 1.46, *P* = 0.294) and OSRS 10% (*F*_1,4_ = 0.14, *P* = 0.723)). This shows that the HTP SSF approach is highly acceptable for rapid screening approaches.

### Trial on a range of steam explosion pretreatments on wheat straw biomass slurries

**Figure 5:**
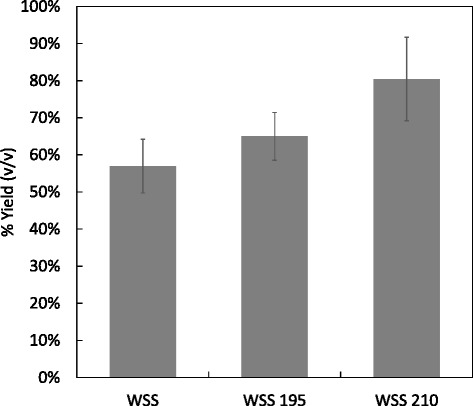
**Trial on a range of steam explosion pretreatments on wheat straw biomass slurries.** Yields based on theoretical ethanol maximum production, wheat straw slurry (WSS) pretreated at 195°C for 10 minutes, 210°C for 10 minutes and with no pretreatment. Sample replicates n = 12.

As a further evaluation of the HTP method and to confirm that quantitative differences in substrate can be detected at a small scale, milled wheat straw was pretreated at different conditions; 195°C for 10 minutes and 210°C for 10 minutes. These materials were then assayed for ethanol production using the 1 mL SSF methodology. The results of 12 replicates (Figure 5) provided an expected increase in final ethanol yield from wheat straw pretreated at 210°C for 10 minutes (80% of theoretical volume), as compared with wheat straw pretreated at 195°C for 10 minutes (64% of theoretical volume).

## Discussion

This work has described a method for enabling the HTP screening of solid substrate materials by SSF. Previous work in the field has developed HTP enzymatic hydrolyses on solid biomass, but SSFs on this material have been carried out only at the laboratory scale; SSF methodology on a solid substrate has not been investigated at the much smaller HTP scale. The SSF methodology introduces the added complexity that any vessels need to be able to withstand gas pressures due to CO_2_ production by the yeast during fermentation whilst being stirred vigorously, hence the choice of screw cap tubes. The choice of caps also had the added benefit of allowing the plate to be stirred whilst on its side, permitting more effective stirring from a standard rotating plate shaker, thus negating the requirement for tailored plate shaking equipment and enabling a better comparison between scales due to uniform shaking.

Additionally, substrate concentrations need to be high enough to allow for measureable ethanol production, but not so high as to inhibit stirring during the SSF incubations. Rapid screening methodology is important to explore the myriad of routes relevant to biofuel production.

One of the main problems of working at such a small scale is the effect that evaporative loss can have on the quantitation of the results [1]. Experiments in the screw cap vessels showed very low loss due to this phenomenon (less than 1% after 72 hours), while other literature results are higher, for example 2.9% after 24 hours in the case of Berlin *et al*. [8], and indeed 7% or less in one hour in the case of Decker *et al*. [16]. These literature results did not utilize screw cap tubes, thus highlighting the benefit of using this kind of closure. Furthermore, with the availability of automated screw cap capper and decapper equipment (Fisher, Loughborough, United Kingdom) it is still highly feasible to use these consumables in HTP screening.

Initial experimentation was carried out to simplify the weighing out of samples, whilst conserving accuracy, as this is a time consuming part of the screening process. Three methods are highlighted: automated weighing, handbill punching and slurry pipetting. The automated method was not pursued as it requires additional equipment not available in most standard laboratories, which can be both expensive and unsuitable for a 96-well system. Handbill-style methodology was investigated using standard Whatman number one FP and OCP. The results showed that a 6-mm diameter disc made with a hole punch gave a highly repeatable weight which could be used at all scales of experimentation. Figure 1 shows that although there were differences in the yields of the individual substrates, as would be expected, there was a continuity of yield percentages across the volumes tested (200 mL, 10 mL and 1 mL), and furthermore there was no statistically significant difference for each substrate (*P* >0.05). This suggests that this methodology would allow for a fast SSF screening of yeast strains with solid cellulose substrates to be carried out. Further to this finding another method of material transfer, slurry pipetting, was also tested. OSRS was again found to have a highly repeatable weight transfer, with standard deviations for a whole plate transfer being 1.7% (w/w) of the mean, although the mean was marginally above the calculated dry weight (DW) of the original slurry. This repeatability would allow for quantitative transfer, should that be necessary, merely by adjusting the original slurry substrate concentration.

An additional and important advantage of using a wet slurry is that it avoids drying the sample, which may effectively reverse the effect of pretreatment [17]. Figure 3 shows the effect of different substrate concentrations on the actual transfer of materials (the small error bars highlighting the high repeatability) better than that achieved by Chundawat *et al*. [4]. Again it can be seen that the pipetted quantity of dry material is marginally higher than the original in the reservoir, but the standard deviation and therefore the repeatability of the transfer is again very good, meaning that should the required substrate concentration be critical, initial slurry dry matter can be adjusted accordingly. This method of weight transfer was then tested at 10 mL and 1 mL scales to establish equivalence both at 2.5% and 10% substrate concentration. The results showed no statistical difference (*P* >0.05), confirming that portioning of material for small scale SSF is equivalent to that for larger scale SSF (Figure 3). It was important to include a substrate concentration at a higher level (10%) to evaluate the suitability of the methodology against substrate concentrations that would more likely be used in a real commercial setting. However, it should also be noted that the method is designed to allow for HTP screening of large numbers of variables, meaning that this initial screen would most certainly be followed up by a more in depth study of any interesting traits found at low substrate concentration.

The 96-well plate format used also simplifies quantification assays, from straightforward colorimetric sugar analyses such as dinitrosalicylic acid (DNS) method [18,19] to full high-performance liquid chromatography (HPLC) analysis [20], as a number of liquid chromatography systems are equipped with 96-well plate autosamplers (Perkin Elmer, Sears Green, United Kingdom). Indeed, sample preparation can be carried out without ever having to leave the plate format, using Acroprep 96-well filters. Where other analysis methods are desired, liquid handling systems can be used to automate a large portion of the preparation, even when 96-well autosamplers are unavailable.

This methodology will therefore allow a flexible technique, enabling HTP analysis using solid substrates for biofuel research, and indeed other applications that require HTP screening on insoluble material. In doing so, the current need to use larger volume shake flasks for these experiments can be eliminated without compromising accuracy. This is particularly important when considering that HTP methodologies are particularly sensitive to experimental variation. For example, when conducting a HTP saccharification analysis of biomass, Oakey *et al*. [21] reported experimental variation as high as 58%, which required considerable statistical control to remedy. This approach allows for the screening of large numbers of permutations in short periods of time, with a high level of repeatability and low laboratory variation, making it ideal for HTP screening.

Wheat straw and oilseed rape straw are important biomass substrates for bioethanol production due to the large tonnages produced. These substrates generally require thermophysical pretreatments in order to be effectively digested to monosaccharides. For this reason, and to further highlight the applicability of the methodology, several pretreatment conditions were further trialed on wheat straw and these samples were then fermented to ethanol using the SSF method using one yeast strain (NCYC 2826). The results shown in Figure 5 demonstrated the effects of the pretreatment on the ethanol yield from each of the pretreated samples. It can be seen that in the case of wheat straw, the yield of ethanol was increased from 64.0 to 80.4% as the pretreatment severity was increased. This set of results shows that trends can be clearly seen in the data from plate scale SSFs. It should be noted that milling can also be considered as a pretreatment and will itself improve the accessibility of enzymes to the lignocellulose, therefore increasing the yield of ethanol [22]. However, as all samples in this part of the study are milled in the same manner, the trend from the steam explosion pretreatment can still be seem clearly. This methodology’s aim is to allow for a fast selection process on a number of different variables that would otherwise be impossible with non-HTP methodologies; the resultant selected samples can then be analyzed in more detail using traditional methods as required.

## Conclusions

This research has practical uses in the biorefining of biomass substrates for second generation biofuels and novel bio-based chemicals. By allowing HTP SSF screening of otherwise recalcitrant lignocellulosic substrates, the method described enables large numbers of inexpensive assays to be carried out quickly and reproducibly in a standard laboratory setting. This will facilitate the rapid screening of substrates, enzymes and fermenting organisms, and the evaluation and optimization of their interactions. Although this methodology looks at relatively low substrate concentrations (up to 10% w/v) it allows for them to be screened quickly, and therefore selections can be made which can then be taken on and studied further at a larger scale.

## Methods

### Materials

M-Real Ep4 OCP (Mason’s Paper, Ipswich, United Kingdom), Whatman number one FP (FP; Fisher Scientific UK Ltd, Loughborough, United Kingdom) pretreated oilseed rape straw slurry (OSRS; *Brassica napus*; Hemp Technology Ltd. Halesworth, United Kingdom) and wheat straw slurry (Dixon Brothers, Rickinghall, United Kingdom) (WSS) were used as the solid substrates in this study. These were representatively chosen to highlight a range of substrates; pure cellulose (FP), highly processed (OCP) and material that requires pretreatment (OSRS and WSS). Circles of 6-mm diameter were made from the OCP and FP using a standard office hole punch (Lyreco, Telford, United Kingdom). The oilseed rape straw and wheat straw were pretreated by steam explosion at a range of severities using a Cambi™ Steam Explosion Pilot Plant (Cambi, Asker, Norway), frozen in liquid nitrogen and cryogenically milled to a fine powder in a 6970EFM Freezer/Mill (two sets of three minutes, Spex Sample Prep, Stanmore, United Kingdom). The resultant biomass was evaluated for dry matter content using a Mettler LP-16 Infrared Dryer Balance (Mettler-Toledo Ltd, Leicester, United Kingdom), and then made into slurries of the desired solid content by adding an appropriate volume of YNB and allowed to hydrate in a continually stirred flask (24 hours).

### Simultaneous saccharification and fermentation at various scales

SSF was performed at 200 mL, 10 mL and 1 mL scales in triplicate. Conical flasks (500 mL) stoppered with a gas-impermeable bung (Cole-Parmer Instrument Co. Ltd, London, United Kingdom) pierced with a 40-mm syringe needle (to allow CO_2_ release) were used to perform the SSF at a scale of 200 mL. A 0.2 μm polyvinylidene difluoride (PVDF) filter (Millipore Corporation, Billerica, Massachusetts, United States) was fixed to the syringe to prevent microbial contamination. For the 10 mL scale, 30 ml universal bottles sealed with metal caps were used (Fisher Scientific UK Ltd, Loughborough, United Kingdom). Two ceramic balls (Pascal Engineering, Crawley, United Kingdom) of 1-cm diameter were used as a stirring aid. Finally, 1.0 mL matrix storage tube plates (Fisher Scientific UK Ltd, Loughborough, United Kingdom) with screw caps were used for SSF at a scale 1 mL, using two 2.5 mm glass beads (Thistle Scientific, Glasgow, United Kingdom) to aid stirring.

A total of 2.5% (w/v) DW of solid sample (OCP, FP and OSRS pretreated at 210°C for 10 minutes) was added at each scale, along with a volume of YNB calculated to ensure final volume after all additions, including yeast and enzyme as required. Samples were then autoclaved and allowed to cool below 30°C before the addition of 20 FPU/g Cellic™ CTec2 cellulase enzyme (Novozymes Corp, Bagsvaerd, Denmark) and 10% (v/v) *Saccharomyces cerevisiae* NCYC 2826 yeast inoculum (2 x 10^8^ cells/mL; NCYC, Norwich, United Kingdom). A further sample was made using Difco YM media (Fisher Scientific UK Ltd, Loughborough, UK) containing 1% (w/v) glucose) 90% v/v and 10% v/v yeast inoculum to see the effect of scale on a standard soluble substrate. Finally controls were made with yeast inoculum and enzyme in YNB to quantify the background levels of ethanol produced in the inocula. All samples were then incubated at 25°C for 72 hours on a single rotating shaker (Infors AG, Basel, Switzerland) set to 180 rpm. Universal bottles of 10 mL volume and 1 mL tubes were placed on their sides to ensure good mixing.

Further SSFs were carried out using 10% (w/v) OSRS; the yeast inoculum and enzyme concentration were kept the same as for the 2.5% (w/v) OSRS with the YNB quantity reduced to ensure the final volume was still 10 mL or 1 mL as appropriate.

### Range of steam exploded pretreated biomass slurries

Oilseed rape straw and wheat straw were both steam exploded at a number of severities (195°C for 10 minutes, 210°C for 10 minutes and no pretreatment) in a Cambi Steam Explosion Pilot Plant. The resultant material was subjected to SSF treatment as described above at 2.5% (w/v) substrate concentration in 1 mL matrix tubes at 25°C for 72 hours. HPLC analysis of supernatant for ethanol production was then conducted.

Ethanol was measured directly by HPLC using Flexar Series LC instrument equipped with a deep-well plate autosampler and a refractive index detector (Perkin Elmer, Seer Green, United Kingdom). Samples were initially heated at 100°C for 5 minutes in a gas-tight tube to stop the enzymatic and fermentation reactions, then filtered through an Acroprep™ 96-filter plate 0.2 μm GHP (hydrophilic polypropylene) membrane (VWR International Ltd, Lutterworth, United Kingdom). The analyses were carried out using an Aminex HPX-87P carbohydrate analysis column (Bio-Rad Laboratories Ltd, Hemel Hempstead, United Kingdom) with matching guard columns operating at 65°C with ultrapure water (Millipore (U.K.) Limited, Watford, United Kingdom) as mobile phase at a flow rate of 0.6 mL/min. The concentration of ethanol was measured against a standard curve of HPLC-grade ethanol (Sigma, Gillingham, United Kingdom).

### Gas chromatography analysis of sugars in solid phase

Original carbohydrate compositions were calculated by hydrolysing substrates to monosaccharides using an adapted version of the Saeman hydrolysis method [23], briefly, 72% (w/w) H_2_SO_4_ at room temperature for three hours, followed by 1 mol/L H_2_SO_4_ at 100°C for two and a half hours. These were then reduced with sodium borohydride (NaBH_4_) and acetylated by addition of 1-methylimidazole and acetic anhydride (Sigma Aldrich, Gillingham, UK) as described in Blakeney *et al*. [24]. The alditol acetates produced from the monosaccharides were then analyzed by gas chromatography using a Perkin-Elmer Autosystem XL (Perkin Elmer, Seer Green, United Kingdom) and a RTX-225 (Restek, Bellefonte, United States) column.

### Liquid handling

Liquid transfer was carried out using a Tecan Freedom Evo™ liquid handling robot equipped with a multi-channel arm (Tecan Group Ltd, Mannedorf, Switzerland). When using slurry, disposable wide bore tips (Starlab (UK), Milton Keynes, United Kingdom) were used to prevent blockages.

## Abbreviations

ANOVA: Analysis of variance
DNS: Dinitrosalicylic acid
DW: Dry weight
FP: Filter paper (Whatman number one)
FPU: Filter paper unit
HPLC: High performance liquid chromatography
GC: Gas chromatography
HTP: High-throughput
NCYC: National Collection of Yeast Cultures
NMR: Nuclear magnetic resonance
OCP: Office copier paper
OSRS: Oilseed rape slurry
PVDF: Polyvinylidene difluoride
SHF: separate hydrolysis and fermentation
SSF: Simultaneous saccharification and fermentation
WSS: Wheat straw slurry
YM: Yeast and mould media
YNB: Yeast nitrogen base
